# Acute psychological responses to official match outcomes in male youth volleyball: an observational repeated-measures study within a single national-level team

**DOI:** 10.3389/fpsyg.2026.1826372

**Published:** 2026-04-22

**Authors:** Marcos Henrique Do Nascimento, Alberto Souza Sá Filho, Vicente Aprigliano, Filipe Manuel Clemente, Carlos Alexandre Vieira, Mário Hebling Campos, Augusto Cezar Rodrigues Rocha, Débora Darck Lopes Costa Arantes, Gustavo Ferreira Pedrosa, Matias Noll, Adam Kawczyński, Lorenzo Laporta, Gustavo De Conti Teixeira Costa

**Affiliations:** 1 Programa de Pós-Graduação em Ciências da Saúde, Faculdade de Medicina, Universidade Federal de Goiás, Goiânia, Brazil; 2 Human Movement and Rehabilitation, Universidade Evangélica de Goiás, Anápolis, Brazil; 3 Graduate Program at the Federal University of Rio de Janeiro, UFRJ, Rio de Janeiro, Brazil; 4 Escuela de Ingeniería de Construcción y Transporte, Pontificia Universidad Católica de Valparaíso, Valparaíso, Chile; 5 Department of Biomechanics, Gdansk University of Physical Education and Sport, Gdańsk, Poland; 6 Applied Research Institute (i2A), Polytechnic University of Coimbra, Coimbra, Portugal; 7 Sport Physical Activity and Health Research & Innovation Center, Coimbra, Portugal; 8 Centro de Educação Física e Desportos, Universidade Federal de Santa Maria, Santa Maria, Brazil; 9 Instituto Federal de Educação, Ciência e Tecnologia Goiano, Ceres, Brazil; 10 Faculty of Medicine, Wrocław University of Science and Technology, Wrocław, Poland

**Keywords:** affective valence, Brunel Mood Scale, ecological validity, Physical Activity Enjoyment Scale, Total Quality Recovery

## Abstract

**Background:**

Official match outcomes may be associated with acute psychological responses in youth athletes, particularly in team sports characterized by high emotional and interpersonal demands.

**Objective:**

To examine acute psychological responses in male youth volleyball athletes during official matches in a national-level competition, focusing on associations with match outcome (win vs. loss), playing status (starter vs. substitute), and assessment moment (pre- vs. post-match).

**Methods:**

Fourteen male athletes (mean age 16.5 ± 0.82 years) from a single Brazilian national-level team were followed across 20 official matches (14 wins and 6 losses). Psychological responses were assessed before and/or after matches using validated instruments: State Anxiety (STAI-IDATE), Brunel Mood Scale (BRUMS), Feeling Scale, Physical Activity Enjoyment Scale (PACES), and Total Quality Recovery (TQR). Generalized estimating equations (GEE) were used with an independent working correlation structure and robust (sandwich) standard errors. Fixed effects included match outcome, playing status, assessment moment, and their interactions. Candidate marginal models were compared via Independence Model Criterion under a parsimonious strategy suitable for small samples.

**Results:**

Losses were associated with increased post-match state anxiety, tension, depression, anger, and confusion, whereas wins were generally associated with stability or more favorable responses in these dimensions. Affective valence also showed an outcome×moment interaction, declining after losses but remaining relatively stable after wins. Vigor demonstrated a three-way interaction between playing status, outcome, and moment, with substitutes showing a more pronounced decline after losses. Fatigue showed main effects of playing status, match outcome, and moment. Enjoyment was higher in wins than losses and higher in starters than substitutes. Perceived recovery showed a playing status×outcome interaction, with starters reporting higher pre-match recovery before matches that resulted in wins.

**Conclusion:**

In this single-team observational sample, acute psychological responses appeared to vary according to match outcome, playing status, and assessment moment. These findings should be interpreted as exploratory associations and may support the practical value of psychological monitoring in youth competitive volleyball.

## Introduction

Competitive youth sport is a multifaceted developmental context that extends beyond technical and tactical execution, imposing physical, cognitive, and emotional demands that can meaningfully shape young athletes’ psychosocial trajectories (Bisagno et al., 2022; Nogueira et al., 2024). During adolescence, marked by neurobiological changes, identity consolidation, and heightened sensitivity to social evaluation, these demands may amplify vulnerability to performance-related stressors (Bisagno et al., 2022; Rossi et al., 2024). In this context, psychological responses associated with competition are central not only for immediate performance, but also for athletes’ sport experience quality and long-term development (Mosqueda et al., 2019; Pierce and Erickson, 2022).

In team sports, psychological responses may be intensified by functional interdependence among teammates and by the shared nature of competitive success and failure, increasing the emotional load associated with matches (Nogueira et al., 2024; Richter and Rother, 2023). Volleyball, in particular, involves rapid sequences of decisive actions, high situational unpredictability, and frequent alternation between success and error, requiring strong psychological readiness and emotion regulation (Richter and Rother, 2023). Under these conditions, even relatively small affective fluctuations may lead to meaningful changes in individual behavior and team dynamics, influencing decision-making, communication, and overall performance during competition (Nogueira et al., 2024).

In youth volleyball, competitive anxiety is a key psychological demand and is commonly conceptualized as multidimensional, encompassing cognitive and somatic components and self-confidence. Elevated cognitive anxiety, characterized by excessive worry and negative thoughts, has been associated with poorer decision-making and increased technical errors in key volleyball actions (Fortes et al., 2019). Recent evidence suggests that competitive anxiety fluctuates across competitive contexts and may be sensitive to match outcomes, particularly in youth athletes. In this regard, psychological resources such as self-efficacy may moderate these responses by attenuating anxiety and supporting more adaptive performance during competition (Nogueira et al., 2024; Rossi et al., 2024).

Beyond anxiety, mood states represent another key domain for understanding competition-related psychological responses and are frequently interpreted through the iceberg profile (del Arroyo-Bosque et al., 2024; Torres et al., 2020). This model suggests that better performance is typically associated with lower tension, depression, anger, fatigue, and confusion, combined with higher vigor (Torres et al., 2020). In youth volleyball, evidence indicates that mood states differ conditionally by competitive outcome, being that losses are associated with increased negative mood and reduced vigor post-match, whereas wins tend to preserve or improve the team’s affective profile (Werneck et al., 2019). Confusion has been linked to cognitive disorganization and impaired emotional processing following adverse competitive events and has been interpreted as a marker of post-match cognitive overload (Chen et al., 2025; Michel-Kröhler et al., 2024).

In addition to negative emotional states, recent research has emphasized the role of positive affective experiences, particularly pleasure and enjoyment, as key indicators of how youth athletes experience competitive sport (Gil-Arias et al., 2020; Mosqueda et al., 2019). Grounded in Self-Determination Theory, accumulated evidence suggests that enjoyment is strongly associated with satisfaction of basic psychological needs (competence, autonomy, relatedness), as well as with engagement and meaning attributed to sport participation (Deci and Ryan, 2000; Mosqueda et al., 2019). In team sports such as volleyball, enjoyment may vary across matches and is sensitive to both assessment timing and match outcome, with wins typically associated with better subjective well-being pre- and post-match and losses linked to less satisfying affective experiences after competition (Werneck et al., 2019).

Playing status may also influence athletes’ psychological responses: reduced predictability of participation, role ambiguity, and lower perceived functional contribution may undermine pleasure, enjoyment, and engagement in the competitive process (Mosqueda et al., 2019). Among substitutes, the combination of limited participation and adverse outcomes may intensify negative psychological responses and reduce the quality of the sport experience, with potential consequences for engagement and retention in youth sport (Purcell et al., 2023; Walton et al., 2024).

Taken together, these psychological domains may be understood as interconnected components of athletes’ acute affective responses to competition. Competitive anxiety and mood states reflect affective dimensions associated with performance-related demands, whereas affective valence and enjoyment capture more immediate experiential evaluations of the competitive context (Mosqueda et al., 2019; Werneck et al., 2019). Perceived recovery, in turn, reflects athletes’ readiness to cope with these demands and may influence how such experiences are processed across matches (Brandão et al., 2019; Nogueira et al., 2015). Rather than representing independent constructs, these dimensions may operate in an integrated manner, jointly reflecting how athletes perceive, interpret, and respond to competitive events (Nogueira et al., 2024; Rossi et al., 2024). Examining these variables simultaneously within an ecological competitive context may therefore provide a more comprehensive understanding of acute psychological responses in youth sport.

Accordingly, the aim of this study was to examine acute psychological responses among male youth volleyball athletes during official national-level competition, using a longitudinal, repeated-measures observational design. Based on previous evidence suggesting that adverse competitive outcomes are associated with less favorable anxiety, mood, and affective responses in youth sport, we expected that psychological responses would vary according to match outcome and assessment moment, with less favorable responses following losses than wins (Nogueira et al., 2024; Werneck et al., 2019). We also expected that playing status could be associated with differences in these responses, particularly under adverse competitive conditions, given that reduced participation certainty and role ambiguity may shape athletes’ emotional experiences in team sports (Mosqueda et al., 2019; Purcell et al., 2023). Considering the observational design, the single-team sample, and the exploratory scope of the study, these expectations were interpreted as theoretically informed but non-confirmatory.

## Materials and methods

### Study design

This study adopted a longitudinal observational design with repeated measures conducted in an ecological competitive context (official national-level matches). Athletes were followed prospectively across a sequence of matches, and psychological responses were assessed before and/or after each match, as detailed below. As no intervention or experimental manipulation was performed, no allocation or blinding procedures were applicable. Reporting was primarily informed by the Transparent Reporting of Evaluations with Nonrandomized Designs (TREND) recommendations, adapted to the present repeated-measures observational monitoring design. Although the study did not involve an intervention, TREND was used to support transparent reporting of participant flow, assessment timing, and repeated outcome monitoring across official matches. The study was approved by the Research Ethics Committee (CAAE: 65290217.2.0000.5083).

### Participants

Fourteen male athletes (mean age 16.5 ± 0.82 years) from a Brazilian national club championship finalist team participated. Although the competition is categorized as U19, team rosters may include younger, eligible athletes. Participants were recruited by convenience sampling from a single Brazilian club team (U19 male category) that reached the national championship finals. The research team approached the coaching staff, and all eligible athletes from the roster were invited to participate; no incentives were offered. All athletes were regularly enrolled in formal education and engaged in systematic, deliberate training (>4 sessions/week/2 h per day). Inclusion criteria were: (i) ≥ 2 years of experience in official volleyball competitions; (ii) absence of injuries or clinical conditions that limited training participation during the data-collection period; and (iii) parental/legal guardian consent. Exclusion criteria were: (i) missing any instrument at any assessment; (ii) absence from any data-collection session; or (iii) absence from more than three training sessions in the 15 days preceding data collection. No participants were lost or excluded after initial screening. Participant flow is summarized as follows: 14 athletes were screened for eligibility, 14 met inclusion criteria, 0 declined participation, and 14 were enrolled and provided consent/assent as applicable. During the monitoring period, all enrolled athletes completed all scheduled assessments. Therefore, analyses were conducted as complete cases for each outcome. A flow diagram is not shown because the study involved a single cohort with complete follow-up and no exclusions after screening.

Playing status (starter vs. substitute) was defined based on the official starting lineup at the beginning of each match (first set). Across all 20 analyzed matches, the same seven athletes (including the libero) consistently composed the starting lineup, whereas the remaining seven athletes were classified as substitutes, in accordance with the competition regulation allowing 14 players per match. Thus, players classified as starters remained starters throughout the monitoring period, and players classified as substitutes remained substitutes, with no within-player variation in playing status across matches. Starters remained on the court for most of the match, whereas substitutes entered only in specific situations (e.g., serving substitutions or occasional 5:1 system adjustments). Consequently, starters accumulated substantially greater playing time than substitutes, although actual playing time was not objectively quantified. Because playing status remained stable across matches for each athlete, inferences related to this factor are based on between-player differences rather than within-player variation and should be interpreted accordingly. Procedures complied with the Declaration of Helsinki. Data were anonymized prior to analysis, and only authorized researchers accessed the dataset.

### Procedures

Data collection took place during the 2024 national competitive season, from June to December, covering 20 official matches. Recruitment and initial screening occurred in June, and follow-up concluded with the last monitored match at the end of December. Athletes were monitored across 20 official matches in national competitions (male U19 category). Matches followed Fédération Internationale de Volleyball rules (2024). In total, 14 wins and 6 losses were recorded (70% win rate).

**Figure 1:**
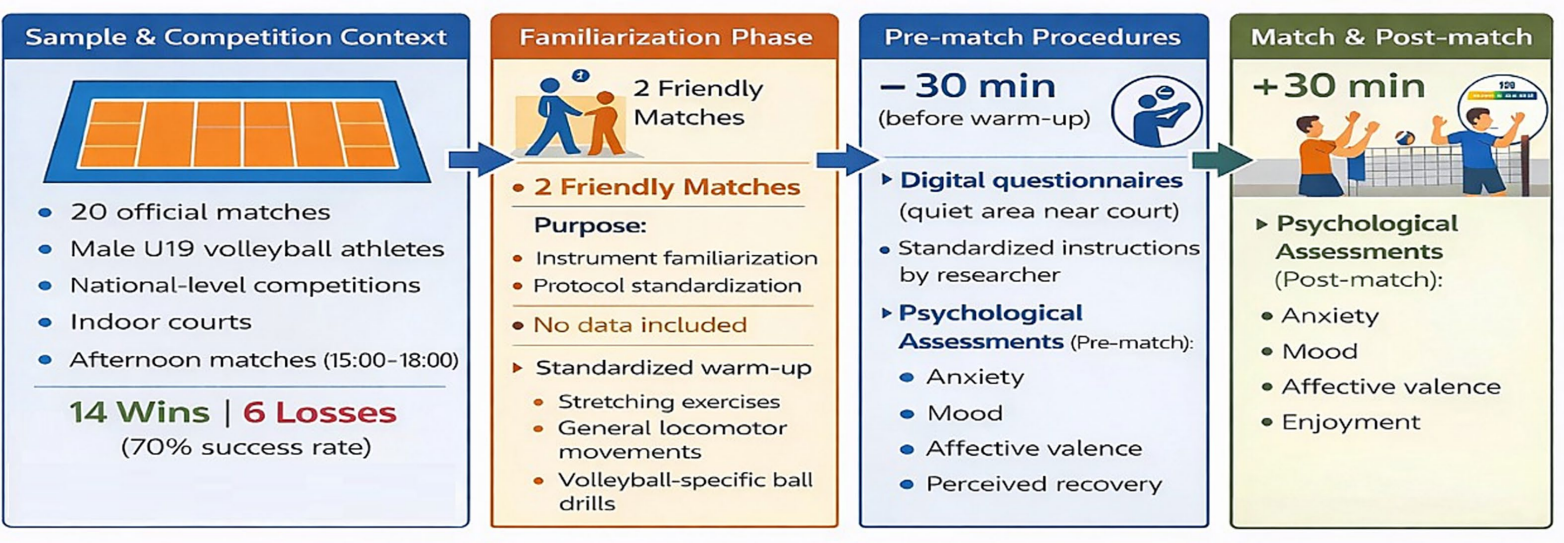
Schematic overview of the study design and data-collection protocol.

Prior to formal data collection, two non-official friendly matches were conducted to familiarize athletes with the assessment instruments. The assessment protocol was standardized: instruments were administered ~30 min before the beginning of pre-match warm-up and ~30 min after match completion. Prior to each match, athletes completed a standardized warm-up comprising stretching exercises, general locomotor movements, and volleyball-specific ball drills (Figure 1).

All matches were played in indoor courts to minimize environmental influences (e.g., wind, precipitation). Matches occurred in the afternoon (15:00–18:00) to reduce potential circadian effects on the assessed responses.

Questionnaires were self-administered individually via a digital platform in a quiet area adjacent to the court. The researcher provided standardized instructions prior to instrument administration and remained available to clarify doubts without interfering with participants’ responses.

Psychological responses were assessed before and approximately 30 min after match completion. Perceived recovery was assessed pre-match, whereas enjoyment was assessed post-match. No adverse events or unintended effects related to the assessment procedures were observed or reported during the study period.

### Variables and instruments

State anxiety: State anxiety was measured with the Brazilian version of the State–Trait Anxiety Inventory (STAI; IDATE), State subscale (Biaggio et al., 1977a). The instrument was originally developed by Spielberger et al. (1970) and later translated and adapted to Brazilian Portuguese following standardized procedures (Biaggio et al., 1977b). Brazilian studies report adequate factorial structure and internal consistency for the IDATE in different populations, supporting its construct validity and reliability (Andrade et al., 2001; Biaggio, 2018; Kaipper et al., 2010).

Mood states: mood states were assessed using the Brazilian 24-item version of the Brunel Mood Scale (BRUMS), derived from the Profile of Mood States (POMS) and adapted for athletes and physically active individuals. The Brazilian BRUMS underwent translation, back translation, and psychometric testing in Brazilian samples, demonstrating satisfactory validity and reliability for detecting altered mood states in athletes (Rohlfs et al., 2008; Rohlfs et al., 2004).

Affective valence: affective valence was assessed using the Feeling Scale (Hardy and Rejeski, 1989), enabling the characterization of the affective response to the exercise stimulus. We adopted a measure with the Feeling Scale (FS), an 11-point bipolar scale (from −5 “very bad” to +5 “very good”) adapted and tested for reproducibility in Brazilian Portuguese, showing acceptable measurement properties for use in exercise contexts (Alves et al., 2019).

Enjoyment: enjoyment was quantified post-session through the Physical Activity Enjoyment Scale (PACES; Moore et al., 2009), which reflects the extent to which the training experience was perceived as pleasant and intrinsically rewarding. PACES, which has been translated, culturally adapted, and shown satisfactory reliability and reproducibility in Brazilian samples engaged in physical activity (Alves et al., 2019; Monteiro et al., 2017).

Perceived recovery (TQR): perceived recovery was measured before each session using the Total Quality Recovery scale, initially proposed by Kenttä and Hassmén (1998) as a 6–20-point scale structurally analogous to Borg’s RPE, in which higher scores indicate better overall recovery and subsequently investigated in Brazilian athletes (Brandão et al., 2019; Nogueira et al., 2015). Studies suggest that TQR is a practical psychometric tool, sensitive to variations in training load and helpful in monitoring recovery state in sports (Brandão et al., 2019; Nogueira et al., 2015).

Playing status: playing status was defined *a priori* based on the official starting lineup for each match. Athletes listed in the starting lineup were classified as starters, whereas all other rostered athletes were classified as substitutes for that match.

### Statistical analysis

#### *Post-hoc* sensitivity analysis

Sample adequacy was evaluated using a *post-hoc* sensitivity analysis, as proposed by Atkinson and Nevill (2001), for a between-subjects design with repeated measures. To evaluate the adequacy of the sample size for detecting effects of interest, sensitivity was expressed as the minimum detectable effect for 80% power (MDE80) on the coefficient scale of the target interaction term(s) from the fitted marginal models. Sensitivity was quantified within the GEE framework using the same specifications adopted for inferential analyses (Gaussian distribution, identity link, independent working correlation, and robust [sandwich] standard errors), with the clustering unit defined as the pre-post pair within each athlete×match.

For outcomes assessed at both pre- and post-match, the target parameter was the three-way interaction (Moment × Match outcome × Playing status). For outcomes assessed at a single time point, sensitivity was evaluated for the two-way interaction (Match outcome × Playing status), as interactions involving moment are not estimable without repeated measurements. Under a two-sided *α* = 0.05, MDE80 was computed using the normal approximation to the Wald test as:

$$\mathrm{MDE}80=(z_{1-\alpha /2}+z_{0.80})\times \mathrm{SE}(\hat{\beta })$$

where $\mathrm{SE}(\hat{\beta })$ is the robust standard error of the relevant interaction coefficient. To facilitate interpretation across outcomes measured on different scales, MDE80 was additionally expressed in standardized units (MDE80_SD) by dividing MDE80 by the empirical standard deviation of each outcome. Sensitivity estimates (MDE80 and MDE80_SD) for each outcome are reported in Supplementary Table S1. Analyses were conducted in R (R Foundation for Statistical Computing, Vienna, Austria) using the geepack package.

#### Primary outcome models

Generalized estimating equations (GEE) were used to estimate conditional differences in psychological responses as a function of match outcome (win vs. loss), playing status (starter vs. substitute), assessment moment (pre vs. post, when applicable), and their interactions. All models used an independent working correlation structure and robust (sandwich) standard errors to prioritize valid inference under repeated measures and small-sample conditions.

Candidate models were pre-specified and compared within each outcome in a parsimonious set that varied (i) the assumed marginal distribution and (ii) the inclusion of interaction terms consistent with the measurement structure. Model fit was evaluated using the Quasi-Likelihood under the Independence Model Criterion (QIC), prioritizing parsimonious specifications suitable for small samples. Model adequacy was further examined through inspection of parameter stability across candidate model specifications and assessment of residual distributions. Sensitivity analyses were conducted using *post-hoc* minimum detectable effect estimates (MDE80) within the GEE framework, providing an indication of the smallest effects that could be reliably detected given the sample size and model structure.

In addition to statistical significance, we quantified the magnitude of conditional differences using standardized contrasts (*Δ*SD), computed as the raw contrast between estimated marginal means (*Δ*) divided by the empirical standard deviation of each outcome (ΔSD = Δ/SD_empirical) calculated from athletes’ responses. To facilitate interpretation among outcomes measured on different scales, ΔSD values were interpreted as standardized mean differences, using conventional benchmarks as heuristic guidance (≈0.2 small, ≈0.5 moderate, ≈0.8 large), while avoiding rigid cut-offs and emphasizing contextual relevance and cross-outcome consistency (Bakeman, 2005; Cohen, 1992; Lakens, 2013). Because repeated-measures designs can yield multiple defensible standardization choices, we used a single, transparent SD_empirical per outcome to support comparability across correlated affective domains and to complement, rather than replace, model-based inference (Morris and DeShon, 2002).

Each athlete contributed repeated observations across 20 matches, with observations nested within athletes over time. Match order was used to define the repeated-measures structure, rather than being included as a fixed effect. The number of observations contributing to each outcome depended on the measurement structure. Outcomes assessed both pre- and post-match contributed two observations per athlete per match, whereas outcomes assessed at a single time point contributed one observation per match. Statistical significance was set at *p* ≤ 0.05, and all inferential analyses were conducted using IBM SPSS Statistics for Windows (version 26.0; IBM Corp). A complete-case approach was adopted to preserve within-subject consistency across repeated measures. Missing data were not imputed because the analytic strategy relied on complete repeated observations within athletes across matches. Therefore, athletes were excluded *a priori* if they missed any assessment point. Although no participants were excluded in the present study and the final analytic dataset contained no missing outcome data, the potential for selection bias associated with this approach is acknowledged as a limitation.

## Results

**Figure 2:**
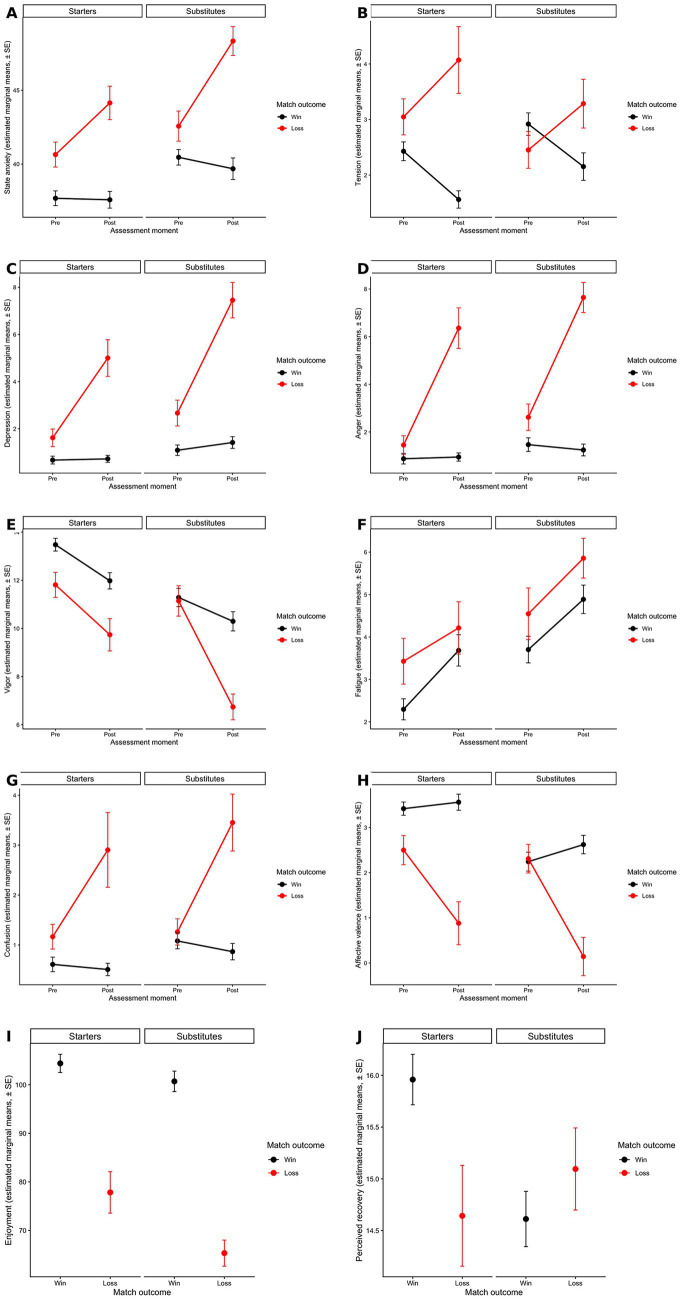
Estimated marginal means (± SE) for psychological outcomes according to match outcome, playing status, and assessment moment. Panels display the estimated marginal means derived from the final GEE models for all study outcomes: **(A)** state anxiety, **(B)** tension, **(C)** depression, **(D)** anger, **(E)** vigor, **(F)** fatigue, **(G)** confusion, **(H)** affective valence, **(I)** enjoyment, and **(J)** perceived recovery. For outcomes assessed pre- and post-match, values are presented according to assessment moment, match outcome, and playing status. For outcomes assessed at a single time point, enjoyment was assessed post-match only and perceived recovery was assessed pre-match only. Error bars represent standard errors.

Table 1 presents observed descriptive means and standard deviations, whereas Figure 2 presents model-based estimated marginal means derived from the final GEE models. Fourteen athletes were included in all analyses and were monitored across 20 official matches. For outcomes assessed both pre- and post-match, two observations per athlete per match were included in the corresponding GEE models, whereas outcomes assessed at a single time point contributed one observation per athlete per match.

**Table 1:** Descriptive data.

Variables		Win (Mean ± SD)				Loss (Mean ± SD)			
		Starters		Substitutes		Starters		Substitutes	
		Pre	Post	Pre	Post	Pre	Post	Pre	Post
State anxiety		37.68±0.63	37.58±0.40	40.64±1.07	44.14±1.94	40.45±0.68	39.68±1.02	42.57±1.36	48.33±1.73
Mood states	Tension	2.42±0.20	1.56±0.12	3.04±0.43	4.07±1.08	2.91±0.11	2.15±0.31	2.45±0.27	3.28±0.51
	Depression	0.67±0.16	0.72±0.20	1.61±0.50	5.00±1.19	1.09±0.15	1.41±0.22	2.66±0.51	7.45±0.97
	Anger	0.87±0.17	0.94±0.25	1.45±0.63	6.35±1.04	1.46±0.34	1.24±0.24	2.61±0.49	7.64±0.74
	Vigor	13.47±0.33	11.97±0.35	11.80±0.75	9.73±1.15	11.28±0.46	10.29±0.50	11.14±0.64	6.73±0.89
	Fatigue	2.29±0.46	3.68±0.57	3.42±0.67	4.21±0.93	3.70±0.21	4.88±0.35	4.54±0.56	5.85±0.48
	Confusion	0.61±0.09	0.51±0.17	1.16±0.36	2.90±1.27	1.08±0.17	0.86±0.18	1.26±0.32	3.45±0.81
Affective valence		3.41±0.20	3.56±0.26	2.50±0.47	0.88±0.72	2.24±0.24	2.62±0.27	2.30±0.41	0.14±0.63
Enjoyment			104.40±1.39		77.83±0.72		100.69±1.61		65.33±3.11
Perceived recovery		15.95±0.28		14.64±0.72		14.61±0.18		15.09±0.39	

To improve clarity and interpretability, the Results section is organized according to the measurement structure of the outcomes: first, outcomes assessed pre- and post-match; second, outcomes assessed at a single time point. The main text focuses on the primary effects and key interaction patterns.

For all outcomes, GEE models were fitted using a Gaussian distribution, identity link, an independent working correlation structure, and robust (sandwich) standard errors. Model fit is reported using QIC for each outcome. However, QIC values should not be directly compared across different outcomes because the variables were measured on different scales.

A multilevel figure displaying estimated marginal means for all psychological outcomes is provided in Figure 2 to facilitate visualization of the main patterns according to match outcome, playing status, and assessment moment.

### Outcomes assessed pre- and post-match

#### State anxiety

The GEE model for state anxiety showed a main effect of playing status and an outcome×moment interaction (QIC_IDATE = 20383.690). Overall, substitutes reported higher anxiety scores than starters (*Δ* = 2.749; *p* < 0.001; ΔSD = 0.41). The outcome×moment interaction indicated distinct temporal patterns according to match outcome: in wins, anxiety remained stable from pre- to post-match (*Δ* = −0.439; *p* = 0.352; ΔSD = −0.07), whereas in losses it increased from pre- to post-match (*Δ* = 4.631; *p* < 0.001; ΔSD = 0.69). In addition, anxiety scores were higher in losses than in wins at both pre-match (*Δ* = 2.536; *p* < 0.001; ΔSD = 0.38) and post-match (*Δ* = 7.605; *p* < 0.001; ΔSD = 1.13), indicating a markedly less favorable post-match profile after defeat.

### Mood states

#### Tension

The GEE model for tension showed a playing status×outcome interaction and an outcome×moment interaction (QIC_Tension = 2858.553). The playing status×outcome interaction indicated that, among starters, tension was higher in matches resulting in losses than in matches resulting in wins (*Δ* = 1.565; *p* < 0.001; ΔSD = 0.66), whereas no significant difference was observed among substitutes (*Δ* = 0.333; *p* = 0.287; *Δ*SD = 0.14). The outcome×moment interaction showed opposite temporal patterns according to match outcome: in wins, tension decreased from pre- to post-match (*Δ* = −0.816; *p* < 0.001; ΔSD = −0.35), whereas in losses it increased from pre- to post-match (*Δ* = 0.929; *p* = 0.030; ΔSD = 0.39). In addition, no difference between wins and losses was observed at pre-match (*Δ* = 0.077; *p* = 0.774; ΔSD = 0.03), whereas post-match tension was higher in losses than in wins (*Δ* = 1.821; *p* < 0.001; ΔSD = 0.77).

#### Depression

The GEE model for depression showed a main effect of playing status and an outcome×moment interaction (QIC_Depression = 4384.776). Overall, substitutes reported higher depression scores than starters (*Δ* = 0.923; *p* < 0.001; ΔSD = 0.57). The outcome×moment interaction indicated that depression remained stable from pre- to post-match in wins (*Δ* = −0.119; *p* = 0.298; ΔSD = −0.07), whereas it increased markedly after losses (*Δ* = 1.274; *p* < 0.001; ΔSD = 0.79). In addition, depression scores were higher in losses than in wins at both pre-match (*Δ* = 0.697; *p* < 0.001; ΔSD = 0.43) and post-match (*Δ* = 2.091; *p* < 0.001; ΔSD = 1.30), indicating a consistently less favorable profile in matches resulting in defeat.

#### Anger

The GEE model for anger showed a main effect of playing status and an outcome×moment interaction (QIC_Anger = 4923.401). Overall, substitutes reported higher anger scores than starters (*Δ* = 0.835; *p* = 0.018; ΔSD = 0.23). The outcome×moment interaction indicated that anger remained stable from pre- to post-match in wins (*Δ* = −0.077; *p* = 0.713; ΔSD = −0.02), whereas it increased markedly after losses (*Δ* = 4.964; *p* < 0.001; ΔSD = 1.37). In addition, anger scores were higher in losses than in wins at both pre-match (*Δ* = 0.862; *p* = 0.025; ΔSD = 0.24) and post-match (*Δ* = 5.903; *p* < 0.001; ΔSD = 1.63), indicating a substantially less favorable post-match profile after defeat.

#### Vigor

The GEE model for vigor showed a playing status × outcome × moment interaction (QIC_Vigor = 7197.406). Among starters, vigor decreased from pre- to post-match in both wins (*Δ* = −1.500; *p* < 0.001; ΔSD = −0.38) and losses (*Δ* = −2.071; *p* = 0.007; ΔSD = −0.52), with higher values in wins than losses at both pre-match (*Δ* = 1.670; *p* = 0.004; ΔSD = 0.42) and post-match (*Δ* = 2.242; *p* = 0.003; ΔSD = 0.57). Among substitutes, vigor also decreased from pre- to post-match in both wins (*Δ* = −0.990; *p* = 0.028; ΔSD = −0.25) and losses (*Δ* = −4.405; *p* < 0.001; ΔSD = −1.11), with no difference between wins and losses at pre-match (*Δ* = 0.143; *p* = 0.846; ΔSD = 0.04) but higher post-match vigor in wins than in losses (*Δ* = 3.558; *p* < 0.001; ΔSD = 0.90). Overall, the most pronounced decline was observed among substitutes after losses. Given the complexity of this three-way interaction and the sample size, this finding should be interpreted cautiously.

#### Fatigue

The GEE model for fatigue showed main effects of playing status, match outcome, and assessment moment, with no significant interactions (QIC_Fatigue = 6162.701). Overall, substitutes reported higher fatigue scores than starters (*Δ* = 1.344; *p* < 0.001; ΔSD = 0.39), fatigue was higher in losses than in wins (*Δ* = 0.869; *p* = 0.010; ΔSD = 0.25), and post-match values were higher than pre-match values (*Δ* = 1.167; *p* < 0.001; ΔSD = 0.34). These findings indicate a consistent pattern of greater fatigue among substitutes, after losses, and following match completion.

#### Confusion

The GEE model for confusion showed an outcome×moment interaction (QIC_Confusion = 2664.116). Confusion remained stable from pre- to post-match in wins (*Δ* = −0.158; *p* = 0.231; ΔSD = −0.07), whereas it increased after losses (*Δ* = 1.964; *p* < 0.001; *Δ*SD = 0.84). In addition, no significant difference between wins and losses was observed at pre-match (*Δ* = 0.367; *p* = 0.080; ΔSD = 0.16), whereas post-match confusion was higher in losses than in wins (*Δ* = 2.490; *p* < 0.001; ΔSD = 1.06). These findings indicate a clearly less favorable post-match profile in matches resulting in defeat.

#### Affective valence

The GEE model for affective valence showed a main effect of playing status and an outcome×moment interaction (QIC_AffectiveValence = 2424.156). Overall, starters reported higher affective valence than substitutes (*Δ* = 0.760; *p* < 0.001; ΔSD = 0.33). The outcome×moment interaction indicated that affective valence remained stable from pre- to post-match in wins (*Δ* = 0.260; *p* = 0.121; ΔSD = 0.11), whereas it decreased after losses (*Δ* = −1.893; *p* < 0.001; ΔSD = −0.82). In addition, no significant difference between wins and losses was observed at pre-match (*Δ* = −0.427; *p* = 0.100; ΔSD = −0.19), whereas post-match affective valence was lower in losses than in wins (*Δ* = −2.580; *p* < 0.001; ΔSD = −1.12). These findings indicate a clearly less favorable post-match affective profile in matches resulting in defeat.

## Outcomes assessed at a single time point

### Enjoyment

The GEE model for enjoyment showed main effects of playing status and match outcome (QIC_Enjoyment = 121413.463). Overall, enjoyment was higher in starters than in substitutes (*Δ* = 11.097; *p* < 0.001; ΔSD = 0.61) and higher in wins than in losses (*Δ* = 7.754; *p* < 0.001; ΔSD = 0.43). Because QIC values are scale-dependent, the magnitude of QIC for enjoyment should not be directly compared with QIC values from other outcomes.

### Perceived recovery

The GEE model for perceived recovery showed a playing status × outcome interaction (QIC_PerceivedRecovery = 1962.789). Among starters, perceived recovery before matches resulting in wins was higher than before matches resulting in losses (*Δ* = 1.316; *p* = 0.016; ΔSD = 0.48), whereas no significant difference was observed among substitutes (*Δ* = −0.483; *p* = 0.313; ΔSD = −0.18). In addition, before matches resulting in wins, starters reported higher perceived recovery than substitutes (*Δ* = 1.347; *p* < 0.001; ΔSD = 0.50), whereas no significant difference was observed before matches resulting in losses (*Δ* = −0.452; *p* = 0.471; ΔSD = −0.17).

## Discussion

This longitudinal, observational study with repeated measures in an ecological context examined acute psychological responses of youth volleyball athletes across 20 official national-level matches, focusing on conditional differences by match outcome (win vs. loss), playing status (starter vs. substitute), and assessment moment (pre vs. post, when applicable). Overall, conditional differences according to outcome and moment were evident for state anxiety, tension, depression, anger, confusion, and affective valence; conditional differences according to playing status and outcome were evident for tension and perceived recovery; and conditional differences according to playing status, outcome, and moment were evident for vigor. Beyond statistical significance, several key win–loss contrasts, particularly at post-match, were large in magnitude across multiple correlated affective domains, including state anxiety, depression, anger, confusion, affective valence, and enjoyment (Bisagno et al., 2022; Rossi et al., 2024). In line with recommendations to interpret standardized effects as heuristic indicators rather than rigid thresholds, emphasis should be placed on the consistency of these patterns across related outcomes (Cohen, 1992; Lakens, 2013). Taken together, these findings suggest that match outcome and playing status were meaningfully associated with acute psychological responses in this specific competitive context, particularly in the post-match period.

State anxiety showed robust conditional differences among outcomes and moments, with a marked worsening after losses. Whereas anxiety remained stable from pre to post in wins (ΔSD = −0.07), it increased substantially after losses (ΔSD = 0.69). Moreover, the loss–win contrast was already present at pre-test (ΔSD = 0.38) and became pronounced at post-test (ΔSD = 1.13), indicating that losses were associated with a large acute shift in anxiety in the post-match period. This pattern aligns with evidence that adolescence is characterized by heightened sensitivity to social evaluation and identity-relevant performance cues, making competitive losses salient stressors (Bisagno et al., 2022; Purcell et al., 2023; Rossi et al., 2024). The consistently higher anxiety in substitutes compared with starters (ΔSD = 0.41) suggests role-related differences in psychological responses, which may reflect lower predictability of participation, reduced perceived control, and heightened social-evaluative concerns in athletes who are less certain about their competitive contribution (Fransen et al., 2022; González-Hernández et al., 2020). Notably, the large post-match loss–win contrast (ΔSD > 1) supports the practical relevance of post-competition monitoring, complementing prior work that often emphasizes primarily pre-competitive anxiety processes (Cerin and Barnett, 2011; Chen et al., 2025).

Across mood states, losses were consistently associated with more maladaptive post-match profiles, and ΔSD values suggest that these conditional differences were not only statistically significant but also substantial. For tension, the win-related reduction from pre to post (ΔSD = **−**0.35) contrasted with the loss-related increase (ΔSD = 0.39), while the post-match loss–win contrast was moderate-to-large (ΔSD = 0.77). These findings are consistent with the view that competitive outcomes modulate affective load in team sports, where shared responsibility and interdependence can intensify emotional responses to adverse results (Nogueira et al., 2024; Richter and Rother, 2023; Werneck et al., 2019). Interestingly, tension displayed role-dependent outcome sensitivity: among starters, tension was substantially higher in losses than wins (ΔSD = 0.66), whereas substitutes showed minimal outcome differences (ΔSD = 0.14). One plausible interpretation is that starters may experience stronger outcome-contingent evaluative pressure because of the centrality of the role and its expectations. In contrast, substitutes may present a more uniformly elevated or role-ambiguous stress profile, reducing the incremental effect of outcome on this specific dimension (Chen et al., 2025; Edgar et al., 2024; Purcell et al., 2023). The higher levels of depression (ΔSD = 0.34) and anger (ΔSD = 0.23) in substitutes relative to starters further reinforce the presence of role-related differences in psychological responses, plausibly shaped by uncertainty regarding participation and perceived contribution (Fransen et al., 2022; Walton et al., 2024). Vigor showed nuanced conditional differences captured by the three-way interaction. Both starters and substitutes displayed pre–post declines in vigor across outcomes, consistent with competitive demands and post-match depletion. However, the decline after losses was especially pronounced among substitutes (ΔSD = −1.11), contrasting with a smaller decline after wins (ΔSD = −0.25). At post-test, substitutes also showed a large win–loss contrast (ΔSD = 0.90). Among starters, pre–post vigor decreases were small to moderate in both wins (ΔSD = −0.38) and losses (ΔSD = −0.52), with moderate win–loss contrasts at pre (ΔSD = 0.42) and post (ΔSD = 0.57). These patterns suggest that the combined context of being a substitute and experiencing a loss may be associated with a disproportionately adverse post-match energetic state, reflecting compounded emotional load, appraisal processes, and uncertainty-related cognitive effort, in addition to physical fatigue (Trecroci et al., 2020; Wu et al., 2024). Although a three-way interaction was observed for vigor, this finding should be interpreted with caution, given the sample size, the number of analyses conducted, and the greater complexity of higher-order interactions. Evidence in volleyball and other team sports supports vigor as a marker of readiness and adaptive affective functioning, which can be disrupted under adverse competitive experiences (Boladeras et al., 2025; Werneck et al., 2019).

Fatigue exhibited main effects without interactions, indicating consistent conditional differences across roles, outcomes, and moments. Substitutes reported higher fatigue than starters (ΔSD = 0.39), losses were associated with higher fatigue than wins (ΔSD = 0.25), and post-match fatigue exceeded pre-match fatigue (ΔSD = 0.34). While higher fatigue in substitutes may appear counterintuitive if interpreted purely as physical load, it is compatible with the notion that perceived fatigue integrates both physical and cognitive-emotional components, including anticipatory stress, uncertainty, and mental fatigue linked to role ambiguity and social-evaluative monitoring (Trecroci et al., 2020; Wu et al., 2024). Findings from competitive settings similarly suggest that adverse outcomes are associated with worse post-match fatigue and well-being profiles (Andrade et al., 2016; Fessi and Moalla, 2018; Horta et al., 2020).

Confusion showed a clear outcome×moment pattern: stability after wins (ΔSD = **−**0.07) versus a substantial increase after losses (ΔSD = 0.84), culminating in a large post-match loss–win contrast (ΔSD = 1.06). This pattern aligns with interpretations of confusion as a marker of post-event cognitive overload and disorganization following adverse competitive experiences, plausibly shaped by discrepancy between expected and achieved outcomes and by rumination on errors and consequences (Chen et al., 2025; Michel-Kröhler et al., 2024). Notably, the post-match contrast exceeding 1 SD supports the practical relevance of confusion as a post-loss monitoring target, given its potential implications for recovery, learning, and subsequent performance preparation.

Affective valence also displayed pronounced outcome-dependent post-match changes. Valence remained relatively stable after wins (ΔSD = 0.11) but declined substantially after losses (ΔSD = **−**0.82), producing a large post-match loss–win contrast (ΔSD = **−**1.12). Moreover, starters reported higher affective valence than substitutes overall (ΔSD = 0.33). This combination suggests that losses not only amplify negative affect but also suppress positive hedonic experience in the immediate post-match window, and that substitutes may be particularly prone to less favorable affective experiences across competitive contexts (Alves et al., 2019; Crane et al., 2023). Given that affective valence is linked to motivational quality and continued engagement, repeated exposure to low-valence post-loss experiences may carry implications for longer-term motivation and retention, especially among athletes with less stable roles (Crane et al., 2023; Ntoumanis et al., 2024).

Enjoyment showed strong conditional differences by outcome and playing status. Starters reported higher enjoyment than substitutes (ΔSD = 0.32), and wins were associated with substantially greater enjoyment than losses (ΔSD = 1.21). The very large win–loss contrast supports the interpretation that match outcome is a dominant correlate of positive psychological responses, while the starter–substitute difference suggests that participation-related experiences (e.g., perceived contribution and social recognition) may shape enjoyment in youth competitions (González-Hernández et al., 2020; Mosqueda et al., 2019; Walton et al., 2024). These findings are consistent with Self-Determination Theory, insofar as satisfaction of competence, autonomy, and relatedness needs, more likely to be reinforced in winning contexts and for athletes with higher participation certainty, supports positive affect and intrinsic enjoyment (Deci and Ryan, 2000; Ntoumanis et al., 2024). From a practical standpoint, the magnitude of the win–loss contrast highlights that post-loss periods may represent high-priority windows for recovery strategies aimed not only at reducing negative affect but also at restoring positive engagement and enjoyment.

Perceived recovery showed role-dependent, outcome-dependent differences. Among starters, recovery before wins was higher than before losses (ΔSD = 0.48), whereas among substitutes, there was no meaningful win–loss difference (ΔSD = **−**0.18). Additionally, before wins, starters reported higher recovery than substitutes (ΔSD = 0.50), while before losses, there was little role difference (ΔSD = **−**0.17). One interpretation is that starters’ pre-match recovery perceptions may be more closely linked to readiness appraisals and confidence associated with favorable competitive trajectories, whereas substitutes’ recovery perceptions may be less coupled to outcome expectations, potentially due to role uncertainty and variability in anticipatory stress (Fransen et al., 2022; Purcell et al., 2023).

Taken together, the pattern of standardized differences indicates that the match outcome was consistently associated with acute psychological responses across multiple affective domains, particularly in the post-match period following losses. Convergent changes were observed for anxiety, negative mood dimensions, confusion, affective valence, and enjoyment, suggesting a coherent pattern of less favorable responses after defeat, with substitutes generally showing less favorable profiles across several outcomes (Bisagno et al., 2022; Rossi et al., 2024). Overall, more consistent patterns were observed for state anxiety, mood disturbances, and affective valence, whereas findings involving higher-order or more specific interaction effects, such as those observed for vigor and perceived recovery, should be interpreted with greater caution due to their analytical complexity and sensitivity to sample size.

## Conclusion

In this longitudinal observational study conducted within a single youth volleyball team, acute psychological responses appeared to vary by match outcome, playing status, and assessment moment. Overall, less favorable responses were observed more often after losses, particularly in the post-match period, whereas wins were generally associated with more stable or favorable patterns. In addition, substitutes tended to present less favorable profiles across several outcomes, especially under adverse competitive conditions. These findings were broadly aligned with the study’s general expectations, although they should be interpreted as context-specific, exploratory, and associative rather than causal. Taken together, the results suggest that psychological monitoring across competitive contexts may be useful in youth volleyball, particularly in the post-match period after losses. Future studies with larger and more diverse samples, objective exposure measures, and broader contextual indicators are needed to test the stability and generalizability of these associations.

## Limitations

First, the small sample size and inclusion of only male athletes limit generalizability to other populations (e.g., female youth volleyball players and athletes at different competitive levels). Second, reliance on self-report may introduce social desirability and interpretation biases, although such measures are widely used in applied monitoring. Third, despite high ecological validity, the longitudinal observational design does not allow causal inference or direct identification of mediating mechanisms. Fourth, objective match exposure (e.g., minutes played) was unavailable, and some conditional differences by starter/substitute status may reflect unmeasured variation in actual participation and role-specific match experiences. In addition, because repeated observations were obtained within the same matches, some responses may also have been influenced by shared match-level contextual factors, such as opponent characteristics, match importance, or collective team dynamics. Although the analytic approach accounted for repeated observations within athletes, the potential for contextual clustering by match should be acknowledged when interpreting the findings. Fifth, the athletes’ socioeconomic backgrounds were not systematically assessed. Socioeconomic context may influence how youth athletes experience competitive pressure and match outcomes, potentially affecting emotional responses and perceived stakes associated with competition. Future research should consider including socioeconomic indicators to better account for these contextual influences. Finally, multiple correlated psychological outcomes and interaction terms were examined, which increases the possibility of unstable or sample-sensitive findings, particularly for higher-order effects. Accordingly, interpretation should emphasize coherent patterns across outcomes rather than isolated statistically significant findings, and ΔSD should be understood as a heuristic indicator of relative magnitude rather than as an instrument-specific clinical threshold (Cohen, 1992; Lakens, 2013; Morris and DeShon, 2002).

## Data Availability

The raw data supporting the conclusions of this article will be made available by the authors, without undue reservation.
